# Preparation of pH-Responsive PET TeMs by Controlled Graft Block Copolymerisation of Styrene and Methacrylic Acid for the Separation of Water–Oil Emulsions

**DOI:** 10.3390/polym17162221

**Published:** 2025-08-14

**Authors:** Indira B. Muslimova, Dias D. Omertassov, Nurdaulet Zhumanazar, Nazerke Assan, Zhanna K. Zhatkanbayeva, Ilya V. Korolkov

**Affiliations:** 1 The Institute of Nuclear Physics, Ibragimov Str. 1, Almaty 050032, Kazakhstan; dias2101@mail.ru (D.D.O.); nurdauletzhumanazar@gmail.com (N.Z.); 2 L.N. Gumilyov Eurasian National University, Satpaev Str., 2, Astana 010000, Kazakhstan; nazerke.asanova65@mail.ru (N.A.); zhatkanbayeva_zhk@enu.kz (Z.K.Z.)

**Keywords:** pH-responsive membranes, track-etched membranes, RAFT graft polymerization, separation, antifouling, poly(ethylene terephthalate)

## Abstract

To develop membranes capable of efficient and switchable emulsion separation under variable pH conditions, pH-responsive surfaces were engineered on poly(ethylene terephthalate) track-etched membranes (PET TeMs) via a two-step UV-initiated RAFT graft polymerization process. Initially, polystyrene (PS) was grafted to render the surface hydrophobic, followed by the grafting of poly(methacrylic acid) (PMAA) to introduce pH-responsive carboxyl groups. Optimized conditions (117 mM MAA, RAFT:initiator 1:10, 60 min UV exposure at 10 cm) resulted in PET TeMs-g-PS-g-PMAA surfaces exhibiting tunable wettability, with contact angles shifting from 90° at pH 2 to 65° at pH 9. Successful grafting was confirmed by FTIR, AFM, SEM, TGA, and TB dye sorption. The membranes showed high degree of rejection (up to 98%) for both direct and reverse emulsions. In direct emulsions, stable flux values (70 ± 2.8 to 60 ± 2.9 L m^−2^ h^−1^ for cetane-in-water and 195 ± 8.2 to 120 ± 6.9 L m^−2^ h^−1^ for o-xylene-in-water) were maintained over five cycles at 900 mbar, indicating excellent antifouling performance. Reverse emulsions initially exhibited higher flux, but stronger fouling; however, flux recovery reached 91% after cleaning. These findings demonstrate the potential of PET TeMs-g-PS-g-PMAA as switchable, pH-responsive membranes for robust emulsion separation.

## 1. Introduction

The evolution of membrane technologies has shifted from traditional passive filtration systems to the development of adaptive, multifunctional platforms capable of responding dynamically to external stimuli [1,2]. This transition is particularly evident in fields such as biomedicine, environmental protection, and industrial filtration, where membrane functionality must extend beyond simple selective separation, in particular for the separation of water-oil emulsions (WOE). Stimuli-responsive membranes, designed to alter their surface properties under external triggers such as pH, temperature, ionic strength, or light, have gained increasing attention for their ability to enhance selectivity, fouling resistance, and operational flexibility [3,4,5].

Track-etched membranes (TeMs) based on poly(ethylene terephthalate) (PET) offer an ideal substrate for the development of such intelligent systems due to their well-defined pore geometry, mechanical robustness, and chemical stability [6,7,8]. Recent advances in controlled polymerization techniques, such as reversible addition–fragmentation chain transfer (RAFT) polymerization, have enabled the precise modification of membrane surfaces, allowing the creation of tailored wettability and switchable separation behavior [9,10]. By grafting functional polymers onto PET TeMs, it is possible to impart dynamic surface characteristics [11,12,13,14].

While conventional PET TeMs primarily enable efficient size-selective filtration [15], current research increasingly emphasizes surface functionality. In particular, strategies have been developed to produce membranes with entirely hydrophilic or hydrophobic surfaces. Radiation-induced grafting of acrylic acid (AA) has been shown to enhance hydrophilicity without compromising mechanical strength, extending usability in filtration, separation, and biomedical applications [16]. Incorporation of poly(1-vinyl-2-pyrrolidone) enables metal nanoparticle immobilization for use in catalytic degradation of pollutants such as metronidazole [17], while grafting of poly(*N*-isopropylacrylamide) via atom transfer radical polymerization (ATRP) introduces temperature responsiveness [18]. Membranes grafted with AA demonstrate high selectivity for alkaline ions (Li^+^, K^+^, Na^+^), valuable for dialysis and ion exchange [19]. On the hydrophobic side, modification with fluorinated monomers like dodecafluoroheptyl acrylate or heptafluorobutyl methacrylate yields hydrophobic membranes (CA > 97°), suitable for membrane distillation and oil–water separation [20,21]. Additionally, the immobilization of hydrophobic vinyl–silica nanoparticles significantly improves water flux and salt separation efficiency in direct contact membrane distillation [22]. The use of lauryl and stearyl methacrylates further enhances oil selectivity, and fluorosilane-based nanoparticles improve antifouling characteristics [23,24]. Thus, targeted functionalization strategies have significantly advanced membranes to selectively interact with specific chemical species or to perform under demanding physical conditions. Nevertheless, in dynamic environments, especially in the treatment of WOE, there remains a growing demand for materials that can reversibly adapt their surface properties.

Among various stimuli-responsive approaches, pH-switchable systems are particularly attractive due to the ease of controlling pH and the availability of polymers with well-defined ionization behavior. Incorporating pH-responsive polymer layers onto PET TeMs expands their applicability to systems where the controlled wettability is required. This responsiveness enables selective permeability, improved antifouling behavior, and potential for self-cleaning under cyclic operation [25,26]. Various pH-responsive polymers have been employed either as individual components or as part of copolymers in the fabrication of smart membranes designed for WOE separation [1,27,28,29]. Poly(acrylic acid) (PAA) and poly(methacrylic acid) (PMAA) are among the most commonly used anionic pH-sensitive polymers. Their carboxyl groups ionize in alkaline media, enhancing membrane hydrophilicity. Membranes grafted with PAA or PMAA typically exhibit separation efficiencies above 90% and maintain fluxes ranging from 1000 to 4000 L m^−2^ h^−1^ depending on emulsion type and structure [30,31,32]. Poly(4-vinylpyridine) (P4VP) offers a basic nitrogen functionality that protonates under acidic conditions, making membranes hydrophilic at low pH [33]. PET-based TeMs grafted with P4VP demonstrated CA variations from 95° (pH 9) to 52° (pH 2), with stable separation efficiencies between 95–100% and fluxes to 7000 L m^−2^ h^−1^ over multiple cycles [34]. Poly(2-(dimethylamino)ethyl methacrylate) (PDMAEMA) and related tertiary amine-containing methacrylates are also used due to their tunable pKa values (~7.3), contributing to superhydrophilic or oleophobic states under mildly acidic or basic conditions. These materials enable flux rates up to 5000 L·m^−2^·h^−1^ and separation efficiencies exceeding 63–99% [35,36,37]. The addition of nanostructures such as SiO_2_ or TiO_2_ can further enhance surface roughness and responsiveness, improving both selectivity and antifouling characteristics under varying pH.

Building on this foundation, PET-based TeMs have emerged as especially favorable substrates for creating pH-responsive forms. In addition to their narrow pore size distribution, mechanical robustness, and chemical inertness to acids. Their suitability for graft polymerization enables precise surface functionalization without compromising pore structure integrity [38].

Our previous studies demonstrated the effectiveness of PET TeMs-g-PS modified with basic P4VP and acidic PAA pH-responsive blocks for emulsion separation; further investigation is needed to understand how the structure of the responsive polymer affects membrane behavior. In this work, PMAA, a structural analog of PAA bearing an additional methyl group, was selected. This feature is expected to increase steric hindrance and hydration capacity, which may influence the membrane’s pH responsiveness and fouling resistance.

In this study, a two-step UV-initiated RAFT graft polymerization strategy at identical testing conditions were employed, the performance of PET TeMs-g-PS-g-PMAA membranes were evaluated. In the first step, a stable hydrophobic polystyrene (PS) layer was grafted onto PET TeMs. In the subsequent step, PMAA was grafted onto the PS chains, introducing pH-sensitive carboxyl groups. This approach allowed controlled grafting while preserving the porosity of the membrane and giving it an adjustable wettability.

The main objectives of this work were to optimize the second-stage grafting conditions, to characterize the physicochemical properties of the modified membranes via FTIR-ATR, SEM, AFM, TGA, and dye toluidine blue (TB) sorption, and to assess membrane performance in the separation of both direct and reverse water-oil emulsions. Special attention was paid to evaluating flux, rejection degree, antifouling properties and flux recovery during multiple filtration cycles. The composition of the permeate and the stability of the feed emulsions were analyzed using ^1^H NMR-spectroscopy and dynamic light scattering (DLS), respectively. These comprehensive evaluations demonstrate the potential of PET TeMs-g-PS-g-PMAA membranes as versatile, responsive platforms for emulsion treatment under variable pH conditions. Generally, the PET TeMs-g-PS-g-PMAA provides useful insights into the role of polymer composition in designing adaptive membrane materials.

## 2. Materials and Methods

### 2.1. Materials and Chemicals

The membrane base included poly(ethylene terephthalate) track-etched membranes (PET TeMs), Mitsubishi Polyester Film. Monomers were styrene (ST) and methacrylic acid (MAA), Sigma-Aldrich. The RAFT agent was 2-(Dodecylthiocarbonothioylthio)-2-methylpropionic acid, Sigma-Aldrich. The photoinitiator was benzophenone (BP), Sigma-Aldrich. Solvents and other chemicals included N,N-Dimethylformamide (DMF), isopropyl alcohol (IPA), chloroform, benzene, o-xylene, sodium hydroxide, and hydrochloric acid, all with analytical grade purity of ≥95%. Deionized water (18.2 MΩ) was prepared using the Akvilon-D 301. UV lamps: OSRAM Ultra Vitalux E27 (315–400 nm, 13.6 W) was used in grafting, and Osram Puritec (254 nm,12 W) was used in photosensitization. Other materials included a polyvinyl chloride (PVC) film and filtration set, ISOLAB Laborgeraete GmbH, and an Ultra-Turrax disperser and Vacstar control pump, IKA.

### 2.2. Preparation and Modification of Track-Etched Membranes

**Figure 1:**
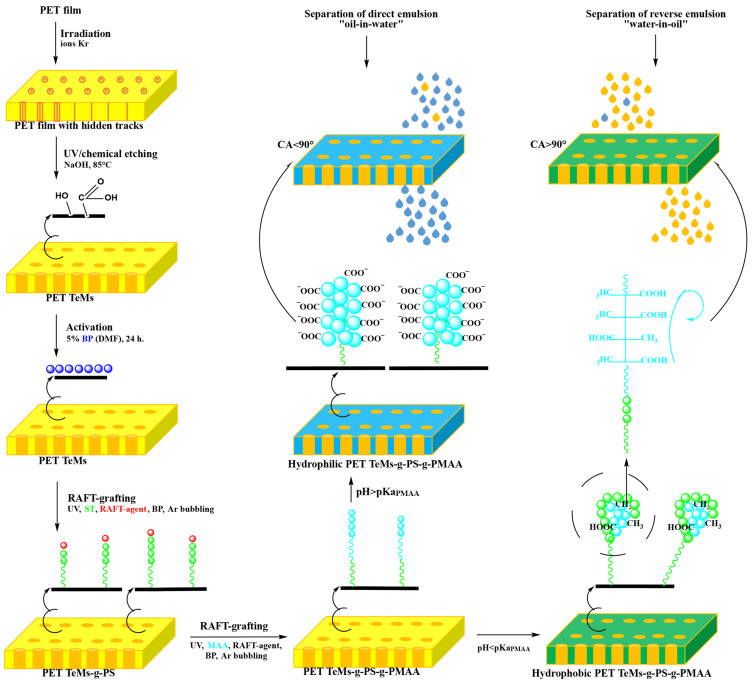
Scheme for the preparation of pH-responsive PET TeMs-g-PS-g-PMAA for the separation of water-oil emulsions.

Figure 1 shows a schematic of the stepwise preparation of pH-sensitive membranes from PET film. This technique was developed on the basis of previous studies on the modification of PET TeMs [24,31,34,39] and optimized to obtain membranes with switchable surface hydrophobicity/hydrophilicity for the separation of water-in-oil/oil-in-water emulsions by changing the pH medium.

#### 2.2.1. Track-Etched Membranes Base Preparation: Irradiation and Etching of PET Films

The production of TeMs was carried out using 23 µm thick poly(ethylene terephthalate) films. These films were subjected to irradiation at the DC-60 cyclotron in Astana, Kazakhstan, employing Kr ions at an energy level of 1.75 MeV per nucleon, achieving a latent track density of approximately 1 × 10^6^ ions/cm^2^. Following irradiation, the films underwent a photosensitization phase under UV light with a 254 nm wavelength, applied from a distance of 10 cm for 30 min on each side. Photosensitization stimulates photooxidation of damaged areas of the irradiated PET film, thereby increasing the chemical track-etching rate, which is significantly slower under normal conditions. Chemical etching was carried out in a solution of 2.2 M sodium hydroxide at an elevated temperature of 85 °C for 10–12 min. As a result of alkaline hydrolysis, the PET ester bonds (—C(=O)O—) were broken, and carboxyl (-COOH) and hydroxyl (-OH) groups were formed on the film surface, and pores with a diameter of about 2.33 ± 0.07 μm were formed in place of the damaged areas. After etching, the PET TeMs were washed in 3% acetic acid solution and in distilled water to neutralize the alkali residue and wash off the hydrolysis products.

#### 2.2.2. UV-Initiated RAFT Graft Polymerization for PET TeMs Modification

The membrane modification was performed via UV-initiated grafting RAFT polymerization using benzophenone (BP) as a UV initiator, 2-(Dodecylthiocarbonothioylthio)-2-methylpropionic acid as a RAFT agent, styrene (ST) and methacrylic acid (MAA) as monomers, and isopropyl alcohol (IPA) as a solvent. Modification of PET TeMs was performed under a UV lamp with a wavelength of 315–400 nm at a power of 13.6 W in an argon atmosphere. To prevent the evaporation of the reaction mixture during UV irradiation, the reaction vessels were covered with PVC film, which also reduces the transmission of short-wavelength UV radiation, thereby minimizing the potential impact of UV exposure on the mechanical properties of the PET TeMs. To activate the surface, PET TeMs were kept in a 5% BP solution in dimethylformamide (DMF) for 24 h without access to light.

Immediately before UV grafting, PET TeMs were washed in DMF to remove excess BP. Then, the PET TeMs were immersed in a container with a reaction mixture of the first monomer—ST. The carboxyl and hydroxyl groups formed after chemical etching, the adsorbed BP on the membrane surface, and the RAFT agent with BP in the reaction mixture ensured uniform grafting of the polymer layer. The grafting of polystyrene (PS) was carried out according to the optimal parameters found in previous research [34]. The concentration of PS in the reaction mixture was 172 mM, the molar ratio of the RAFT agent–initiator in the reaction mixture was 1:10, the distance from the UV source was 7.5 cm, and the UV irradiation time was 60 min. The grafted PS layer, with DG_PS_ 2.5–2.7% (calculated as described in Equation (1)), provided stable hydrophobicity on the surface of PET TeMs-g-PS with a CA 98 ± 3°; unmodified PET TeMs are moderately hydrophilic, with a CA of 60°. The pore structure of the hydrophobic PET TeMs-g-PS was preserved, with a slight decrease in pore diameter from 2.33 ± 0.07 to 2.14 ± 0.1 μm.

In the second step, a similar procedure was used for grafting MAA onto the surface of PET TeMs-g-PS with optimization of the parameters for controlled wettability. The concentration of methacrylic acid varied between 30 and 350 mM, the molar ratio of the RAFT agent–initiator was from 1:1 to 1:30, the distance from the UV source was from 7.5 to 15 cm, and the UV irradiation time was from 30 to 60 min. Grafting of poly(methacrylic acid) (PMAA) was achieved by activating the dormant radical of the RAFT agent at the ends of the TeMs-g-PS PET chains, which ensured controlled growth of the second block [40,41,42]. The selection of optimal parameters for grafting pH-sensitive PET TeMs-g-PS-g-PMAA was carried out based on the response of the CA of their surface to changes in the acidity of the medium at pH 2 and pH 9.

### 2.3. PET TeMs-g-PS-g-PMAA Characterization

#### 2.3.1. Contact Angle (CA) Measurement and pH-Responsivity Evaluation

The CA of the membrane surface with liquids was measured at six different positions on the sample using a digital microscope with 1000× magnification, employing the static drop method at room temperature. CA measurements showed variation within ±3°. The surface free energy (ω, mN/m) and its polar component (γ_p_, mN/m) were calculated by the Owens, Wendt, Rabel, and Kelble method using diiodomethane as a non-polar liquid. The pH responsivity of PET TeMs-g-PS-g-PMAA was evaluated by measuring the CA response under different pH conditions to identify the optimal grafting parameters of PMAA that resulted in the most pronounced transition in wettability between hydrophilicity and hydrophobicity.

The transition in wettability was observed at pH values below and above the pKa_(PMAA)_ 4.8. Figure 1 visualizes the controlled transition of membrane surface wettability in response to a change in pH. The membranes were immersed in solutions with pH values from 1 to 9 for 30 min prior to CA measurement. pH values above 9 were not studied, to avoid degradation of the PET TeMs’ backbones.

#### 2.3.2. Degree of Grafting (DG) Determination

The degree of grafting (DG, %) was calculated by measuring the weight of the membranes before and after the grafting process, according to the following Equation (1):

(1) $$DG=\left(m_{2}-m_{1}\right)/m_{1}\times 100\%$$

where $m_{1}$ is the mass of the membrane before grafting, and $m_{2}$ is the mass of the membrane after grafting.

#### 2.3.3. Atomic Force Microscopy (AFM)

AFM on an NT-206 device (ALC Microtestmachines, Gomel, Belarus) was used to evaluate the surface morphology of the modified membranes. The parameters of average roughness (Ra, nm) and root-mean-square roughness (Rq, nm), as well as local mechanical properties such as adhesion force (Fa, nN) and elastic modulus (E, MPa) were studied. Measurements were carried out in five scanning zones (4 × 4 μm) with subsequent data processing in Surface Explorer software 4.2.

#### 2.3.4. UV-Vis Spectroscopy

A Specord-250 UV-vis spectrophotometer (Analytik Jena, Jena, Germany) was used to analyze the presence of carboxyl groups on the membrane surface before and after modification. The concentration of carboxyl groups was determined based on the sorption of toluidine blue (TB) dye followed by measurement of its desorption at a wavelength of 633 nm.

#### 2.3.5. Thermogravimetric Analysis (TGA)

TGA was performed on a Pyris 1 TGA device (PerkinElmer, Waltham, MA, USA) in the temperature range from 0 to 700 °C at a heating rate of 10 °C/min under nitrogen atmosphere, to study the thermal stability of the membranes and to determine the decomposition temperatures and composition of the PET TeMs-g-PS-g-PMAA polymer layers.

#### 2.3.6. Fourier Transform Infrared Spectroscopy (FTIR)

FTIR spectra were recorded on an InfraLUM FT-08 spectrometer (Lumex, Saint-Petersburg, Russia) using an ATR attachment to analyze the chemical structure of the membranes and to confirm the presence of functional groups introduced during the grafting process. A resolution of 2 cm^−1^ with a minimum number of scans of 20 was used for the analysis. The spectra were processed using the SpectraLUM^®^ software 2.0.1.295 suite. Peak intensities were normalized relative to reference peaks at 1409 and 1245 cm^−1^, corresponding to phenyl ring vibrations (C–H bending coupled with ring stretching) and the asymmetric stretching vibration of C(=O)–O, respectively.

#### 2.3.7. Scanning Electron Microscopy (SEM)

SEM was used to investigate the surface morphology of the membranes and to monitor pore diameter, using a Hitachi TM 3030 instrument (Hitachi, Tokyo, Japan).

### 2.4. Performance Assessment of PET TeMs-g-PS-g-PMAA in Water-Oil Emulsion Separation

The performance of membranes with an area of 0.001256 m^2^ was tested using a dead-end vacuum filtration unit shown in Figure S1, which was employed in previous studies [39]. Vacuum pressure of 900, 700, or 500 mbar was maintained using a Vacstar control pump. Depending on the emulsion type, the membranes were soaked for 30 min in water at pH 2 for reverse emulsions and at pH 9 for direct emulsions (Figure 1). The emulsions were prepared by mixing oil and water components at a volume ratio of 1:100 using an Ultra-Turrax homogenizer at 24,000 rpm for 1 min. The oil components included o-xylene, chloroform, and cetane. Water at pH 2 was used as the dispersed component for reverse emulsions, while water at pH 9 was used as the external component for direct emulsions.

Membrane performance was evaluated by calculating the flux (F) of the filtered liquid using Equation (2), as previously described in earlier works [24,31,34,39]. The rejection degree (R) was calculated using a modified approach adapted from reference [43], as shown in Equation (3):

(2) F=V/(S×t)

(3) $$R=\left(1-\left(\frac{Cp}{Cf}\right)\right)\times 100\%$$

where F is the flux, L m^−2^ h^−1^; V is the volume of external component that permeates through the membrane, L; S is the filtration area of PET TeMs-g-PS-g-PMAA, m^2^; t is the flow time, h; R is the rejection degree, %; Cp, Cf are the concentrations of the dispersed phase in the permeate and feed, vol.% (i.e., oil in direct emulsions or water in reverse emulsions). The feed emulsions were prepared with a fixed dispersed phase concentration of 1 vol.%, allowing Cf to be considered constant across all tested systems.

#### 2.4.1. Evaluation of PET TeMs-g-PS-g-PMAA Fouling and Flux Recovery in Water-Oil Separation

Membrane fouling and flux reduction performance were evaluated by calculating the flux recovery and total flux reduction factor according to Equations (4) and (5) [31,43]:

(4) $$FR=(F_{2}/F_{1})\times 100\%$$

(5) $$TR=(1-(F_{0}/F_{1}))\times 100\%$$

where FR is the flux recovery, %; TR is the total flux reduction factor, %; $F_{1},\;F_{2}\;$ are the fluxes of external components determined before and after emulsion separation, L m^−2^ h^−1^; $F_{0}$ is the emulsion separation flux, L m^−2^ h^−1^.

The pure external component flux was measured during reverse emulsion separations of chloroform, cetane, and o-xylene, as well as during direct emulsion separations with water at pH 9. After each separation, the membranes were cleaned by soaking in a pH 2 solution for reverse emulsions and in a pH 9 solution for direct emulsions. The flux of the external component was then measured again.

#### 2.4.2. Analytical Techniques for Emulsion and Permeate Characterization

Cp were determined using quantitative ^1^H NMR spectroscopy. Spectra were acquired on a Spinsolve 80 MHz benchtop spectrometer (Magritek, Aachen, Germany) using the 1D EXTENDED+ pulse sequence with 16 scans, 6.4 s acquisition time, 15 s repetition time, and 90° pulse angle. Calibration curves were constructed by correlating the integral areas of characteristic peaks (4.823 ppm for water, 7.344 for chloroform, 1.165 for cetane, 1.540 for o-xylene) with known volume concentrations (vol.%). The concentration of the dispersed component in the permeate was calculated based on the integral values after dispersion using a homogenizer, as no visible droplets remained in the collected permeate.

The droplet size distribution of the prepared emulsions was analyzed using a NanoBrook 90Plus PALS analyzer (Brookhaven Instruments, Nashua, NH, USA) based on dynamic light scattering (DLS). Emulsions were dispersed prior to analysis to ensure uniformity. The measurement was carried out at 25 °C in disposable polystyrene cuvettes with a fixed scattering angle of 90°. For each emulsion type, a single sample was analyzed continuously over 40 min using 20 consecutive scans to monitor potential changes in droplet size.

## 3. Results and Discussions

PET TeMs-g-PS with a stable hydrophobic layer (DG_PS_ of 2.6 ± 0.1%, CA of 98 ± 3°, pore diameter of 2.33 ± 0.07 µm) was used as a substrate for UV-initiated RAFT graft polymerization of PMAA to create pH-responsive membranes. As shown in Figure 1, the Z-group (-SC(CH_3_)_2_COOH) of RAFT-agent, which “sleeps” at the PS chain ends, activates the grafting of PMAA to the PS-g-PET TeMs. The reaction pathways for the formation of block copolymer on the surface of the PET TeMs are well described in an earlier work [31].

**Figure 2:**
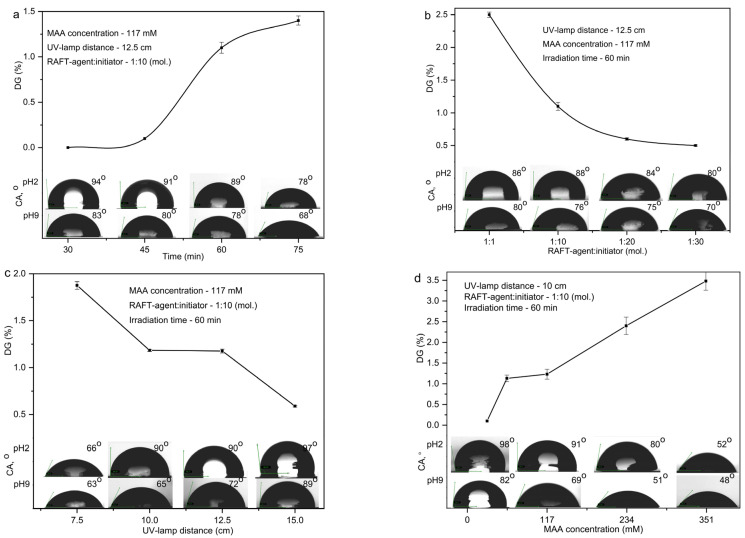
CA at pH 2 and pH 9 and corresponding DG_PMAA_ for PET TeMs-g-PS-g-PMAA obtained under different UV-initiated RAFT graft polymerization conditions: time (**a**), molar ratio of RAFT-agent:initiator (**b**), distance from UV-lamp (**c**) and concentration of MAA (**d**).

Figure 2 presents the changes in DG_PMAA_ and CA of the PET TeMs-g-PS-PMAA surface under varying polymerization conditions. Increasing the grafting time from 30 to 75 min, raising the MAA concentration from 30 to 350 mM, and reducing the distance to the UV source from 15 to 7.5 cm resulted in an increase in DG_PMAA_ from 0 to 3.48 ± 0.22%. The closer proximity to the UV source enhanced UV intensity, while longer exposure promoted more efficient radical formation and monomer conversion. As a result, a clear dependence of grafting degree and wettability on UV exposure conditions was observed; higher DGPMAA values corresponded to a greater concentration of carboxyl groups and stronger hydrophilization, with CA decreasing from 98° to 52° at pH 2. At pH 9, even membranes with a minimal DG_PMAA_ of 0.1% exhibited hydrophilicity with CA < 90°. These trends are consistent with known UV-dose-dependent photopolymerization behavior and agree with previous reports [44,45], which also reported correlation between the quantification of DG_PMAA_, CA, and the polymerization conditions of hydrophilic monomers. The mechanical properties of the membranes remained largely stable at longer UV distances but decreased significantly at the shortest distance: 15 cm—340 kPa, 12.5 cm—350 kPa, 10 cm—270 kPa, and 7.5 cm—60 kPa. However, at all tested distances, the membranes retained sufficient strength for further use in filtration processes.

Increasing the molar ratio of the RAFT agent to the initiator in the reaction mixture from 1:1 to 1:30 caused a decrease in the DG_PMAA_ from 0.5 to 2.5 ± 0.4% (Figure 2b). This trend is attributed to the quantitative predominance of the initiator, which diminished the control exerted by the RAFT agent and promoted the formation of PMAA homopolymer rather than grafted polymer chains on the PET TeMs’ surface. Furthermore, the pH responsivity of these membranes, as determined by contact angle (CA) measurements (Figure 2b), changed by only 6–12° when the pH was varied from 2 to 9, suggesting uncontrolled polymerization and premature chain termination. Under such conditions, a broader molecular weight distribution leads to an uneven distribution of pH-sensitive functional groups, thereby attenuating the overall pH response [46,47].

**Figure 3:**
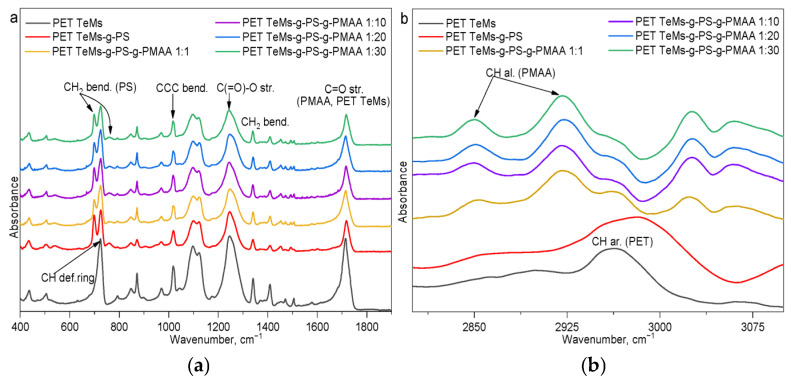
FTIR-ATR spectra of PET TeMs, PET TeMs-g-PS, and PET TeMs-g-PS-g-PMAA obtained at different RAFT agent:initiator molar ratios in the ranges 400–1800 cm^−1^ (**a**) and 2800–3100 cm^−1^ (**b**).

FTIR-ATR spectra (Figure 3) of the membranes before and after grafting consisted of characteristic PET TeMs-g-PS absorption peaks, and they agreed on the wave numbers in the articles [48,49]. For the PET TeMs, the absorption peaks for the ester C=O groups were 1712 cm^−1^, for aromatic ring bending CH 1409 cm^−1^, bending CCC 1017 cm^−1^, stretching CC 872 cm^−1^; for bending CH_2_ groups 1340 cm^−1^, for stretching C(=O)-O groups 1245 cm^−1^, for stretching C-O groups 971 cm^−1^. The absorption peaks of PS for out-of-plane C-H bending were 700 cm^−1^ and 760 cm^−1^, for aromatic C=C stretching, the values were 1601 cm^−1^, 1492 cm^−1^, and 1452 cm^−1^. After PMAA grafting, notable spectral changes appeared, as clearly seen in Figure 3b, with the emergence of absorption bands at 2852 cm^−1^ and 2925 cm^−1^ corresponding to the aliphatic C-H vibrations of PMAA. The carboxyl (-COOH) and hydroxyl (-OH) groups characteristic of PMAA were not distinctly registered in the FTIR spectra due to their relatively low concentration. However, the sorption of TB dye confirmed the presence of terminal -COOH groups on the PET TeMs-g-PS-g-PMAA surface. As the DG_PMAA_ increased from 0.1 to 3.48 ± 0.22%, the COOH groups’ concentration slightly increased from 0.95 to 1.16 mmol/L (0.8 mmol/L for PET TeMs-g-PS [31]).

A more detailed analysis of the FTIR peak ratios is presented in Table 1 and shows that as the molar ratio of the RAFT-agent to the initiator increases from 1:1 to 1:30, the intensity ratios I_1712_/I_1409_ and I_1712_/I_1245_ decrease from 5.00 to 4.00 and from 0.91 to 0.82, respectively, indicating a reduction in the number of carboxyl (-COOH) groups. Simultaneously, the intensity ratios I_2850_/I_1409_ and I_2925_/I_1409_ increase from 0.17 to 0.43 and from 0.33 to 0.57, respectively, which is consistent with the formation of a more branched polymer architecture and hindered packing of polymer chains. These findings confirm that an excess of initiator leads to uncontrolled polymerization, reducing grafting efficiency, decreasing the number of available -COOH sites, and consequently resulting in decreased pH responsivity of the membranes.

**Figure 4:**
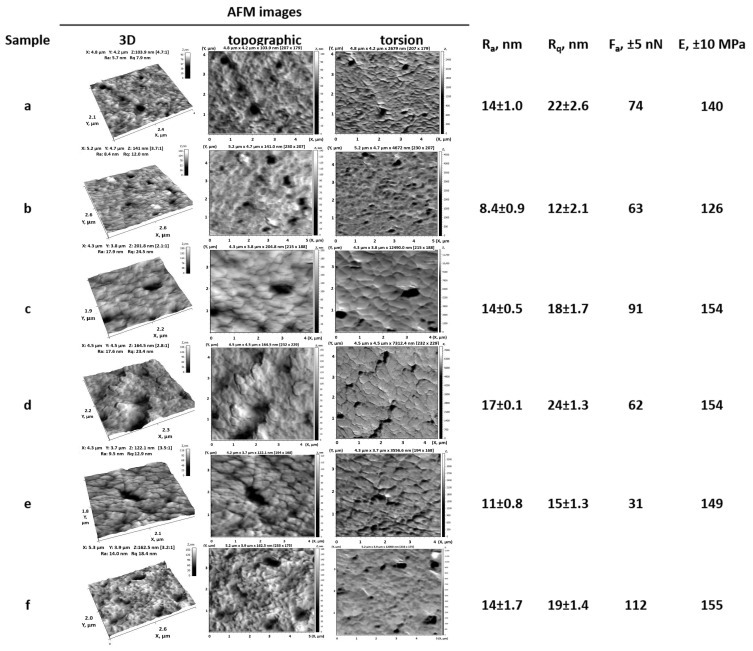
3D, topographic, and torsion AFM images and basic surface characteristics of PET TeMs (**a**), PET TeMs-g-PS (**b**), and PET TeMs-g-PS-g-PMAA obtained using different irradiation times: 30 min (**c**), 45 min (**d**), 60 min (**e**), and 75 min (**f**). All samples were prepared with a constant PMAA concentration of 117 mM, RAFT agent–initiator molar ratio of 1:10, and a UV-source distance of 10 cm.

According to the obtained AFM images (Figure 4), as the irradiation time increases from 30 to 75 min, the grafting of PMAA onto the PET TeMs-g-PS surface (Ra = 8.4 ± 0.9 nm, Rq = 12 ± 2.1 nm, E = 126 ± 10 MPa, Fa = 63 ± 5 nN) leads to the formation of surface irregularities, making the morphology more pronounced and complex. The Ra and Rq values increase to 17 ± 0.1 and 24 ± 1.3 nm, respectively, while the mechanical resistance to deformation increases, as evidenced by an increase in the E to 155 ± 10 MPa.

Significant changes in interfacial adhesion between the PMAA layer and the substrate were observed. For samples grafted for 30 and 75 min, the Fa value increased to 91 and 112 ± 5 nN, respectively. However, for samples irradiated for 60 min, Fa decreased to 31 ± 5 nN and fluctuated within the measurement error at 62 ± 5 nN for samples grafted for 45 min.

**Figure 5:**
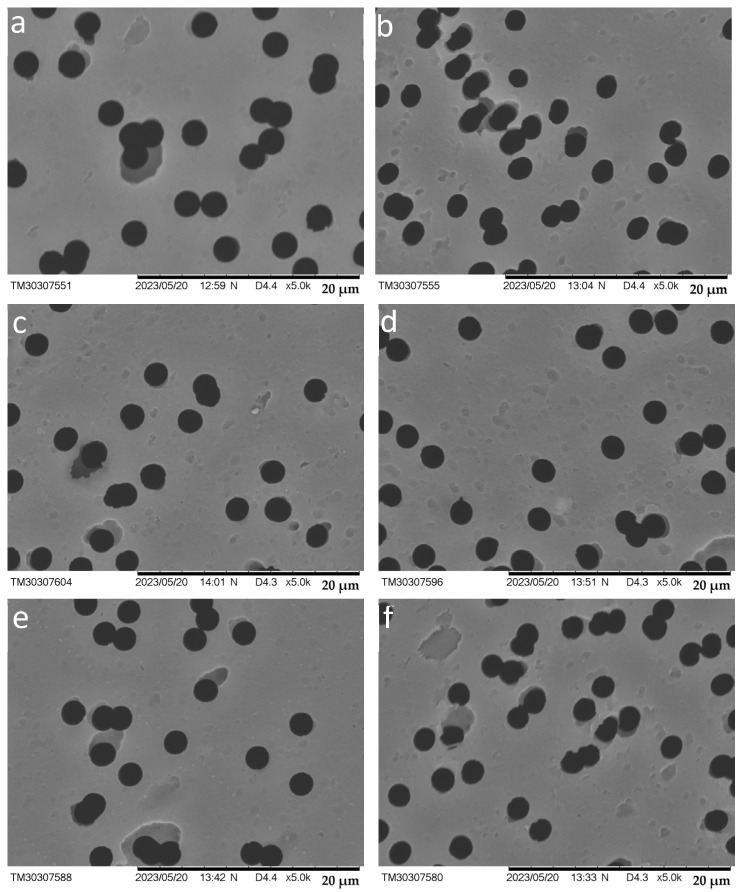
SEM images of PET TeMs (**a**), PET TeMs-g-PS (**b**), and PET TeMs-g-PS-g-PMAA obtained at different distances from the UV-source: 15 cm (**c**), 12.5 cm (**d**), 10 cm (**e**), and 7.5 cm (**f**). All samples were prepared with a constant PMAA concentration of 117 mM, RAFT agent-to-initiator molar ratio of 1:10, and an irradiation time 60 min.

SEM images before and after PMAA grafting are presented in Figure 5. As DG_PMAA_ increases, the surface morphology becomes smoother at the microscale. A decrease in the distance between the UV source and the sample from 15 to 7.5 cm results in a reduction in pore diameter from 2.09 ± 0.08 µm to 1.90 ± 0.08 µm due to denser PMAA grafting (DG_PMAA_ increases from 0.59 ± 0.01% to 1.87 ± 0.04%) onto the PET TeMs-g-PS surface (initial pore diameter 2.14 ± 0.16 µm).

**Figure 6:**
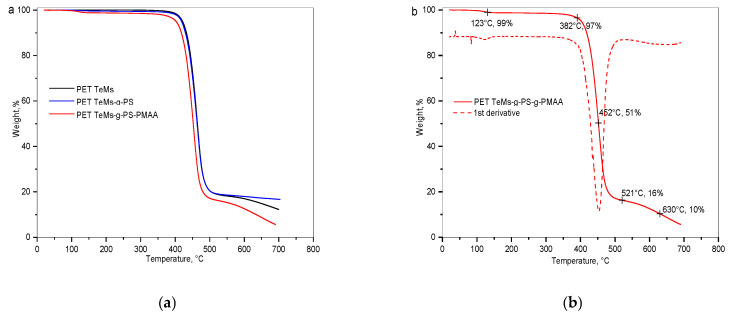
TGA thermograms (**a**) of PET TeMs, PET TeMs-g-PS, and PET TeMs-g-PS-g-PMAA; TGA and corresponding DTG curves (**b**) of PET TeMs-g-PS-g-PMAA.

TGA and DTG curves for PET TeMs-g-PS-g-PMAA membranes are shown in Figure 6. The thermogram of the pristine PET TeMs exhibits a single-phase decomposition process characteristic of the main PET polymer backbone. Decomposition started at 382 °C, with the maximum degradation rate observed at 463 °C. PS grafting led to a slight reduction in thermal stability, with the decomposition temperature remaining close to that of the pristine PET TeMs. The data for PET TeMs-g-PS are consistent with earlier work [31]. PMAA grafting resulted in more significant changes; the first peak at 123 °C, with a 1% mass loss, indicates a physical evaporation process, as hydrophilic PMAA side groups can retain water or small amounts of volatile compounds, such as the IPS solvent. The main decomposition process shifted to 452 °C, with a mass loss of 51%, indicating PMAA chain degradation.

Thus, PS and PMAA grafting is confirmed by thermogravimetric analysis, demonstrating trends similar to those observed with PAA, but with a more pronounced shift in decomposition temperatures and greater mass loss. Additional degradation peaks at 521 °C and 630 °C, with mass losses of 16% and 10%, respectively, may be associated with further thermal degradation of membrane residues.

As a result of this study, pH-responsive PET TeMs-g-PS-g-PMAA membranes were obtained under optimal conditions of UV-initiated controlled RAFT graft polymerization of PMAA onto the hydrophobic PET TeMs-g-PS surface, as follows: PMAA concentration of 117 mM, RAFT agent-to-initiator molar ratio of 1:10, irradiation time of 60 min, and a UV-source distance of 10 cm. Under these conditions, the pore diameter was 2.01 ± 0.03 μm, the DG_PMAA_ reached 1.1 ± 0.06%, and the membrane surface exhibited a pronounced pH response; at pH 9, the CA was 65°, while at pH 2, it increased to 90°. To further confirm the pH-responsive behavior of PET TeMs-g-PS-g-PMAA membranes, additional CA measurements were performed across the pH range of 1–9, using membranes prepared under optimal grafting conditions. As shown in Figure S3, the CA was 91° at pH 1 and gradually decreased to 62° at pH 9. This trend reflects the progressive deprotonation of carboxylic acid groups in the PMAA blocks and confirms the presence of pH-responsive functionality on the membrane surface. These results further validate the successful PMAA grafting and support the selection of pH 2 and pH 9 as representative switching points corresponding to the hydrophobic and hydrophilic states, respectively, for use in the emulsion separation experiments.

PMAA grafting was confirmed by FTIR-ATR spectroscopy, where the spectra after modification exhibited absorption bands characteristic of aliphatic CH groups of PMAA (2852 and 2925 cm^−1^), along with a decrease in the intensity of PET and PS peaks. Thermogravimetric analysis revealed an additional mass loss at 123 °C, attributed to the evaporation of adsorbed volatile components, and a shift in the main decomposition process to 452 °C, with a 51% mass loss, indicating PMAA chain degradation. AFM and SEM analyses revealed an increase in surface roughness and the formation of a denser and more uniform morphology as DG_PMAA_ increased, accompanied by changes in surface mechanical properties.

**Figure 7:**
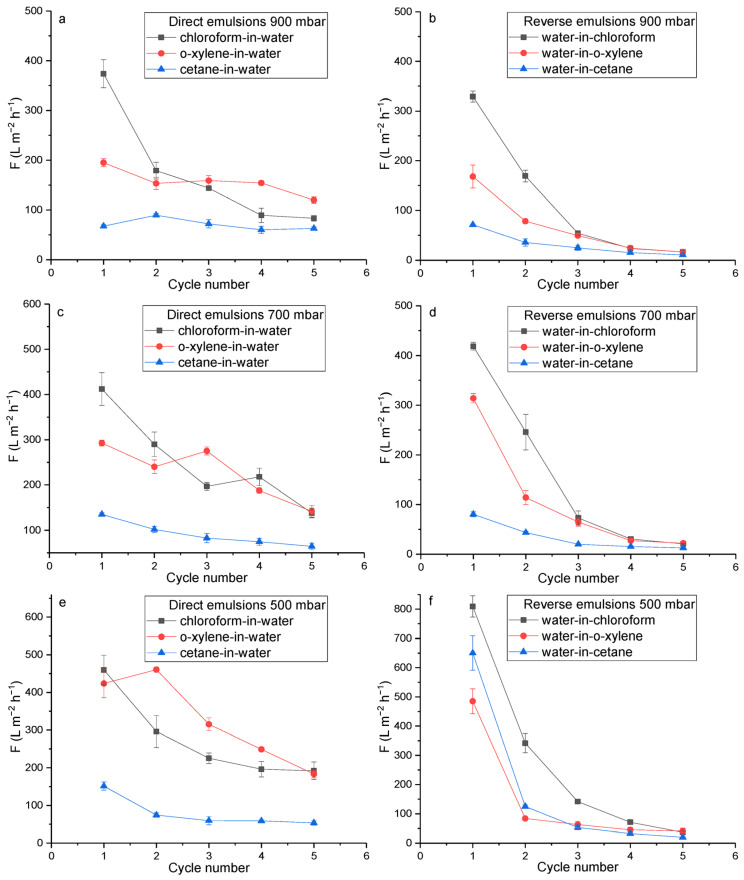
F variation during five separation cycles of direct (**a**,**c**,**e**) and reverse (**b**,**d**,**f**) emulsions at 900 mbar (**a**,**b**), 700 mbar (**c**,**d**), and 500 mbar (**e**,**f**), using PET TeMs-g-PS-g-PMAA.

The obtained PET TeMs-g-PS-g-PMAA were tested for the separation of direct and reverse two-component WOE. Since the most distinct wettability transitions were observed between pH 2—CA 90° and pH 9—CA 65° (Figure S3), these values were selected as representative points for evaluating the switchable behavior of the membranes in emulsions. The emulsions remained visually stable and turbid throughout the test (Figure S4), with droplet sizes ranging from 0.4 to 8.6 μm over 5–40 min, as confirmed by DLS measurements (Table S1, Figure S5). Despite some coalescence over time, all systems maintained droplet sizes within the range generally accepted for macroemulsions (1–100 μm), supporting their classification as emulsions. For the separation of “oil-in-water” emulsions, the membrane was converted to a hydrophilic state by pre-soaking for 30 min in pH 9, allowing water to pass freely through the pores while oil droplets were retained. Conversely, for reverse “water-in-oil” emulsions, the membrane was made hydrophobic (pH 2), enabling the oil phase to pass through while dispersed water droplets were retained. Figure 7 shows the flux dynamics of PET TeMs-g-PS-g-PMAA over 5 separation cycles of various emulsion types at vacuum pressures of 500, 700, and 900 mbar. Table 2 summarizes the quantitative parameters for the first cycle at 900 mbar, including flux (F, L m^−2^ h^−1^), flux recovery ratio (FR, %), total flux reduction ratio (TR, %), rejection degree over five cycles (R, %), and comparison with other studies.

F gradually decreased over five cycles for all types of WOE. For direct emulsions, PET TeMs-g-PS-g-PMAA showed high initial F and over 90% of the oil phase was separated from the water. For example, at 900 mbar, the initial F for the chloroform-in-water emulsion was 370 ± 28 L m^−2^ h^−1^ with R of 89%. For less volatile and more viscous oils, F was lower: for o-xylene in water, 195 ± 8.2 L m^−2^ h^−1^ and R 91%; for cetane in water, 70 ± 2.8 L m^−2^ h^−1^ and R 98%. R was calculated based on quantitative ^1^H NMR spectroscopy, using the integral intensities of water and oil signals (Figure S2), since no emulsion droplets were detected in the permeate by DLS.

Lowering the pressure from 900 to 700 or 500 mbar increased the vacuum, and F significantly rose in the first cycle. For chloroform in water, F increased from 370 ± 28 to 410 ± 36 L m^−2^ h^−1^, for o-xylene in water from 195 ± 8.2 to 420 ± 37 L m^−2^ h^−1^, and for cetane in water from 70 ± 2.8 to 150 ± 11 L m^−2^ h^−1^. However, a higher vacuum led to a more pronounced F drop over cycles. For chloroform-in-water at 900 mbar, F decreased to 80 ± 5.6 L m^−2^ h^−1^, and at 500 mbar to 190 ± 24 L m^−2^ h^−1^. A similar trend was observed for other emulsion types. Stable F over 5 cycles was noted for cetane-in-water. At low vacuum (900 mbar), F slightly decreased from 70 ± 2.8 to 60 ± 2.9 L m^−2^ h^−1^. Increasing the vacuum to 700 and 500 mbar led to a drop from 135 ± 3.6 to 65 ± 7.3 L m^−2^ h^−1^ and from 150 ± 11 to 50 ± 5.9 L m^−2^ h^−1^, respectively. This indicates that for direct emulsions, the membrane shows better stability under lower vacuum, despite a lower initial F, and the membrane shows better stability at higher pressure (900 mbar) where vacuum suction is weaker. In contrast, lower pressure (500 mbar) results in stronger suction, which accelerates membrane fouling by drawing more oil droplets into the pores. As a result, flux drops more rapidly over repeated cycles. SEM images of the membrane before and after completing all separation tests (including both hydrophilic and hydrophobic operation modes) revealed the development of surface fouling and partial pore blockage (Figure S6). These changes are associated with the observed decline in flux, especially under low-pressure, high-suction conditions.

The value of F for reverse emulsions (Figure 7b,d,f) decreased more sharply than for direct emulsions (Figure 7a,c,e), indicating surface and pore fouling by organic components. At 900 mbar, F for water in chloroform dropped from 330 ± 10 to 17 ± 3.4 L m^−2^ h^−1^, for water in o-xylene from 170 ± 23 to 17 ± 0.9 L m^−2^ h^−1^, and for water in cetane from 70 ± 1.4 to 11 ± 2.6 L m^−2^ h^−1^. Under a stronger vacuum, F in the first cycle was even higher compared with direct emulsions. For water in chloroform at 500 mbar, F was 810 ± 37 L m^−2^ h^−1^, at 700 mbar 420 ± 7.7 L m^−2^ h^−1^, at 900 mbar 330 ± 10 L m^−2^ h^−1^. However, in all cases, F dropped sharply by cycles 3–5, down to 40, 20, and 17 L m^−2^ h^−1^, respectively. A similar pattern was seen for water in o-xylene, where F dropped to 40–20 L m^−2^ h^−1^ by cycle 5. The lowest F was observed for water in cetane, at 500 mbar from 650 ± 58 to 20 ± 2 L m^−2^ h^−1^, at 700 mbar from 80 ± 5.1 to 10 ± 2.2 L m^−2^ h^−1^, and at 900 mbar from 70 ± 1.4 to 11 ± 2.6 L m^−2^ h^−1^. Moreover, at 500 mbar in cycles 4–5, water leakage through the membrane was detected, and R for water in cetane dropped to 80%, while in other modes, it exceeded 85%. Despite the sharp F decrease for reverse emulsions, the rejection degree remained high at 80–93%.

Thus, PET TeMs-g-PS-g-PMAA shows more stable hydrophilicity than hydrophobicity, confirmed by more stable F over five separation cycles and by more pronounced organic fouling in the hydrophobic mode. Additionally, for chloroform in water, FR was 85% and TR was 2%, indicating 85% recovery of initial F and very low irreversible fouling (2%). For water in chloroform, initial F was recovered at 91%, but irreversible fouling reached 25%. This difference was due to the nature of the fouling; in hydrophilic mode, oil droplets are weakly adsorbed and easily washed away, while in hydrophobic mode, organic components deposit firmly in the pores and are harder to remove.

Comparison with PET TeMs-g-PS-g-PAA, studied in our previous work [31], shows that using poly(acrylic acid) (PAA) enables a more efficient transition to the hydrophobic state than PMAA under similar grafting method and testing conditions. For PET TeMs-g-PS-g-PAA at pH 2, a higher F of 1400 L m^−2^ h^−1^ was achieved, due to the better ability of PAA to collapse at low pH, while PMAA forms less compact coils due to conformational hindrance [45,48] and overlaps the PS layer. This confirms that the choice of polar block significantly affects wettability switching efficiency. PET TeMs-g-PS-g-PMAA, in terms of key parameters, is comparable and even superior to M-PD/HPA@PVDF and Ti_2_SnC-MAX-PES in antifouling and separation performance, but slightly inferior to superhydrophilic and superhydrophobic MIL88A@TA@APTES-PVDF and PAN@Co-MOF. Despite the lower flux compared with some high-performance superhydrophilic membranes, PET TeMs-g-PS-g-PMAA provides stable F in direct emulsion separation, maintains high R (89–98%), and exhibits effective antifouling properties in both hydrophilic (chloroform in water, TR 2%) and hydrophobic (water in cetane, TR 10%) configurations. The versatility toward emulsion type and controllable wettability make these membranes promising for applications requiring switchable separation modes.

## 4. Conclusions

As a result of UV-initiated RAFT graft polymerization of MAA onto the surface of PET TeMs-g-PS, PET TeMs-g-PS-g-PMAA pH-responsive membranes were obtained. The optimized grafting conditions (117 mM PMAA, RAFT–initiator 1:10, 60 min, 10 cm from UV source) provided DG_PMAA_ of 1.1%, pH response CA 65° (pH 9)—90° (pH 2), and a pore diameter of 2.01 µm.

The grafting of PMAA was confirmed by several analytical methods. New bands appeared in the FTIR spectra at 2852 and 2925 cm^−1^, characteristic of CH-group vibrations of PMAA. TGA showed additional thermal decomposition stages at 521 °C and 630 °C, and the main degradation peak shifts from 463 °C to 452 °C, indicating the presence of the grafted polymer. SEM revealed smoothing of the surface morphology and pore narrowing. AFM detected an increase in surface roughness Ra and Rq from 8.4 nm and 12 nm (PET TeMs-g-PS) to 17 and 24 nm, respectively. TB sorption results showed an increase in the content of carboxyl groups from 0.8 to 1.16 mmol/L.

Membrane performance was assessed using direct and reverse model emulsions. Emulsion stability and droplet size distribution were confirmed by DLS measurements, demonstrating sub-10 µm droplet sizes throughout filtration. After filtration, no droplets were detected in the permeate, even after redispersion. The rejection degree (R) was evaluated based on ^1^H NMR quantification of trace components in the permeate, providing a reliable assessment even in the absence of droplets by DLS data. PET TeMs-g-PS-g-PMAA demonstrated high efficiency in the separation of direct emulsions: stable F over five cycles, rejection degree of 80–98%, and low irreversible fouling (TR up to 2% for “chloroform-in-water”). In reverse emulsions, higher initial F values were observed, but with a significant decrease already by cycles 3–5, indicating less stable hydrophobicity at low pH.

Thus, despite limited hydrophobic stability, PET TeMs-g-PS-g-PMAA membranes exhibit excellent antifouling properties, high rejection degree, and versatility due to their pH responsivity. These membranes can be considered a competitive solution among switchable membrane systems for emulsion separation. Future research prospects relate to improving the membrane stability at low pH and enhancing performance in the hydrophobic state.

## Figures and Tables

**Table 1:** Intensity ratios of FTIR-ATR absorption bands for PET TeMs, PET TeMs-g-PS, and PET TeMs-g-PS-g-PMAA obtained at different RAFT agent–initiator molar ratios.

Sample	I_2850_/I_1409_	I_2850_/I_1245_	I_2925_/I_1409_	I_2925_/I_1245_	I_1712_/I_1409_	I_1712_/I_1245_
PET TeMs	-	-	-	-	4.91	0.93
PET TeMs-g-PS	-	-	-	-	1.67	0.31
PET TeMs-g-PS-g-PMAA RAFT agent–initiator 1:1, DG_PMAA_ = 2.5%	0.17	0.03	0.33	0.06	5.00	0.91
PET TeMs-g-PS-g-PMAA RAFT agent–initiator 1:10, DG_PMAA_ = 1.1%	0.20	0.03	0.40	0.06	5.40	0.84
PET TeMs-g-PS-g-PMAA RAFT agent–initiator 1:20, DG_PMAA_ = 0.6%	0.18	0.03	0.35	0.06	4.83	0.88
PET TeMs-g-PS-g-PMAA RAFT agent–initiator 1:30, DG_PMAA_ = 0.5%	0.43	0.09	0.57	0.12	4.00	0.82

**Table 2:** Main quantitative separation parameters for direct and reverse emulsions using PET TeMs-g-PS-g-PMAA, compared with the literature data.

Material	Property	Emulsion	Pressure	F, L m^−2^ h^−1^	R, %	FR, %	TR, %	Ref.
PET TeMs-g-PS-g-PMAA	Hydrophilicity pH > pKa_PMAA_	Chloroform in water	900 mbar	370 ± 28	89	88	2	This work
			700 mbar	410 ± 36	90	-	-	
			500 mbar	460 ± 39	90	-	-	
		o-Xylene in water	900 mbar	195 ± 8.2	91	69	52	
			700 mbar	290 ± 9.3	92		-	
			500 mbar	420 ± 37	91		-	
		Cetane in water	900 mbar	70 ± 2.8	98	54	64	
			700 mbar	135 ± 3.6	97		-	
			500 mbar	150 ± 11	95		-	
	Hydrophobicity pH < pKa_PMAA_	Water in chloroform	900 mbar	330 ± 10	92	91	25	
			700 mbar	420 ± 7.7	90		-	
			500 mbar	810 ± 37	93		-	
		Water in o-xylene	900 mbar	170 ± 23	90	81	19	
			700 mbar	310 ± 9.8	91			
			500 mbar	485 ± 43	90			
		Water in cetane	900 mbar	70 ± 1.4	83	70	10	
			700 mbar	80 ± 5.1	85			
			500 mbar	650 ± 58	80			
PET TeMs-g-PS-g-PAA	Hydrophilicity pH > pKa_PAA_	Chloroform in water	900 mbar	2500	94 ± 5	82	22	[31]
	Hydrophobicity pH < pKa_PAA_	Water in chloroform	900 mbar	1400	97 ± 1	96	46	
Ti2SnC-MAX-PES	Hydrophilicity	Oil in water	0.3 kPa	355	80	65	-	[50]
MIL88A@TA@APTES-PVDF	Superhydrophilicity	Oil in water	Gravity	2600	99	94	-	[51]
PAN@Co-MOF	Superoleophobicity, water wetting	Cyclohexane in water	2–8 kPa	1600	99	-	-	[52]
	Superhydrophobicity, oil wetting	Water incyclohexane	2–8 kPa	1040	99	-	-	
M-PD/HPA@PVDF	Hydrophilicity	Hexane in water	0.3 kPa	150	99	83	37	[53]

## Data Availability

The original contributions presented in the study are included in the supplementary material, further inquiries can be directed to the corresponding author.

## Supplementary Materials

The following supporting information can be downloaded at: https://www.mdpi.com/article/10.3390/polym17162221/s1, Figure S1: Dead-end vacuum filtration system used for membrane testing; Figure S2: ^1^H NMR calibration curves for determining dispersed phase concentrations (Cp) in permeate, used for calculating membrane rejection degree (R) in emulsion separation; Figure S3 CA of PET TeMs-g-PS-g-PMAA (DGPMAA 1.1±0.06%) as a function of pH (1–9); Table S1:Stability of the emulsions before separation; Figure S4: Pictures of emulsions during time (before filtrations); Figure S5: DLS results for each emulsion (stability before separation during 5 min to 40 min with the step of 105 sec); Figure S6: SEM images of PET TeMs-g-PS-g-PMAA membranes before (a) and after (b) five separation cycles showing the development of surface fouling.

## References

[1] Zhang N., Yang X., Wang Y., Qi Y., Zhang Y., Luo J., Cui P., Jiang W. (2022). A Review on Oil/Water Emulsion Separation Membrane Material. J. Environ. Chem. Eng..

[2] Liu X., Zhang G., Al Mohawes K.B., Khashab N.M. (2024). Smart Membranes for Separation and Sensing. Chem. Sci..

[3] Karunakar K.K., Cheriyan B.V., Anandakumar R., Murugathirumal A., Senthilkumar A., Nandhini J., Kataria K., Yabase L. (2025). Stimuli-Responsive Smart Materials: Bridging the Gap between Biotechnology and Regenerative Medicine. Bioprinting.

[4] Rehman A., Sohail M., Baig N., Yuan K., Abdala A., Wahab M.A. (2025). Next-Generation Stimuli-Responsive Smart Membranes: Developments in Oil/Water Separation. Adv. Colloid. Interface Sci..

[5] Gao Z., Dong Y., Huang C., Hussain Abdalkarim S.Y., Yu H.Y., Tam K.C. (2025). On-Demand plus and Minus Strategy to Design Conductive Nanocellulose: From Low-Dimensional Structural Materials to Multi-Dimensional Smart Sensors. Chem. Eng. J..

[6] Korolkov I.V., Mashentseva A.A., Güven O., Gorin Y.G., Kozlovskiy A.L., Zdorovets M.V., Zhidkov I.S., Cholach S.O. (2018). Electron/Gamma Radiation-Induced Synthesis and Catalytic Activity of Gold Nanoparticles Supported on Track-Etched Poly(Ethylene Terephthalate) Membranes. Mater. Chem. Phys..

[7] Chakarvarti S.K. (2009). Track-Etch Membranes Enabled Nano-/Microtechnology: A Review. Radiat. Meas..

[8] Barsbay M., Güven O. (2014). Grafting in Confined Spaces: Functionalization of Nanochannels of Track-Etched Membranes. Radiat. Phys. Chem..

[9] Hadi M.K., Wang X., Peng Y., Sangaraju S., Ran F. (2024). Functional Polymeric Membrane Materials: A Perspective from Versatile Methods and Modification to Potential Applications. Polym. Sci. Technol..

[10] Semsarilar M., Abetz V., Semsarilar M., Abetz V. (2021). Polymerizations by RAFT: Developments of the Technique and Its Application in the Synthesis of Tailored (Co)Polymers. Macromol. Chem. Phys..

[11] Rong L.H., Caldona E.B., Advincula R.C. (2022). PET-RAFT Polymerization under Flow Chemistry and Surface-initiated Reactions. Polym. Int..

[12] Adamson A.W., Klerer J. (2015). Modification of Pet Surfaces with End-Functionalized Polymers Prepared from Raft Agents to Achieve Antibacterial Properties = Modifikasi Permukaan Pet Dengan Polimer-Polimer Fungsional Dari Agen Raft Untuk Mencapai Sifat Antibakteri. J. Electrochem. Soc..

[13] Pan K., Ren R., Li H., Cao B. (2013). Preparation of Dual Stimuli-Responsive PET Track-Etched Membrane by Grafting Copolymer Using ATRP. Polym. Adv. Technol..

[14] Yeszhanov A.B., Korolkov I.V., Shakayeva A.K., Lissovskaya L.I., Zdorovets M.V. (2023). Preparation of Poly(Ethylene Terephthalate) Track-Etched Membranes for the Separation of Water-Oil Emulsions. Eurasian J. Chem..

[15] Tai S.L., Abidin M.N.Z., Ma’amor A., Hashim N.A., Hashim M.L.H. (2025). Polyethylene Terephthalate Membrane: A Review of Fabrication Techniques, Separation Processes, and Modifications. Sep. Purif. Technol..

[16] Parmanbek N., Aimanova N.A., Mashentseva A.A., Barsbay M., Abuova F.U., Nurpeisova D.T., Jakupova Z.Y., Zdorovets M.V. (2023). e-Beam and γ-rays Induced Synthesis and Catalytic Properties of Copper Nanoclusters-Deposited Composite Track-Etched Membranes. Membranes.

[17] Parmanbek N., Sütekin S.D., Barsbay M., Aimanova N.A., Mashentseva A.A., Alimkhanova A.N., Zhumabayev A.M., Yanevich A., Almanov A.A., Zdorovets M.V. (2023). Environmentally Friendly Loading of Palladium Nanoparticles on Nanoporous PET Track-Etched Membranes Grafted by Poly(1-Vinyl-2-Pyrrolidone) via RAFT Polymerization for the Photocatalytic Degradation of Metronidazole. RSC Adv..

[18] Friebe A., Ulbricht M. (2007). Controlled Pore Functionalization of Poly(Ethylene Terephthalate) Track-Etched Membranes via Surface-Initiated Atom Transfer Radical Polymerization. Langmuir.

[19] Smolinska K., Bryjak M. (2014). Plasma Modified Track-Etched Membranes for Separation of Alkaline Ions. Open Access J. Sci. Technol. AgiAl Publ. House.

[20] Shakayeva A.K., Yeszhanov A.B., Zhumazhanova A.T., Korolkov I.V., Zdorovets M.V. (2024). Fabrication of Hydrophobic PET Track-Etched Membranes Using 2,2,3,3,4,4,4-Heptafluorobutyl Methacrylate for Water Desalination by Membrane Distillation. Eurasian J. Chem..

[21] Shakayeva A.K., Yeszhanov A.B., Borissenko A.N., Kassymzhanov M.T., Zhumazhanova A.T., Khlebnikov N.A., Nurkassimov A.K., Zdorovets M.V., Güven O., Korolkov I.V. (2024). Surface Modification of Polyethylene Terephthalate Track-Etched Membranes by 2,2,3,3,4,4,5,5,6,6,7,7-Dodecafluoroheptyl Acrylate for Application in Water Desalination by Direct Contact Membrane Distillation. Membranes.

[22] Korolkov I.V., Kuandykova A., Yeszhanov A.B., Güven O., Gorin Y.G., Zdorovets M.V. (2020). Modification of PET Ion-Track Membranes by Silica Nanoparticles for Direct Contact Membrane Distillation of Salt Solutions. Membranes.

[23] Guo Z., Wang Y., Liang Z., Zhang Z., Xie J., Gui X., Hou B., Mo D., Lu L., Yao H. (2023). Hydrophobic Modified PET Ion Track-Etched Membrane for Oil/Water Separation. J. Water Process Eng..

[24] Yeszhanov A.B., Muslimova I.B., Melnikova G.B., Petrovskaya A.S., Seitbayev A.S., Chizhik S.A., Zhappar N.K., Korolkov I.V., Güven O., Zdorovets M.V. (2022). Graft Polymerization of Stearyl Methacrylate on PET Track-Etched Membranes for Oil–Water Separation. Polymers.

[25] Al-Shaeli M., Benkhaya S., Al-Juboori R.A., Koyuncu I., Vatanpour V. (2024). PH-Responsive Membranes: Mechanisms, Fabrications, and Applications. Sci. Total Environ..

[26] Maaz M., Elzein T., Bejjani A., Barroca-Aubry N., Lepoittevin B., Dragoe D., Mazerat S., Nsouli B., Roger P. (2017). Surface Initiated Supplemental Activator and Reducing Agent Atom Transfer Radical Polymerization (SI-SARA-ATRP) of 4-Vinylpyridine on Poly(Ethylene Terephthalate). J. Colloid. Interface Sci..

[27] Dansawad P., Yang Y., Li X., Shang X., Li Y., Guo Z., Qing Y., Zhao S., You S., Li W. (2022). Smart Membranes for Oil/Water Emulsions Separation: A Review. Adv. Membr..

[28] Gupta R.K., Dunderdale G.J., England M.W., Hozumi A. (2017). Oil/Water Separation Techniques: A Review of Recent Progresses and Future Directions. J. Mater. Chem. A Mater..

[29] Rasouli S., Rezaei N., Hamedi H., Zendehboudi S., Duan X. (2021). Superhydrophobic and Superoleophilic Membranes for Oil-Water Separation Application: A Comprehensive Review. Mater. Des..

[30] Wu H., Wang Y., Mao X., Gao Z., Luo S., Kipper M.J., Huang L., Tang J. (2024). Recent Advances in Electrospinning Smart Membranes for Oil/Water Separation. Surf. Interfaces.

[31] Muslimova I.B., Zhumanazar N., Melnikova G.B., Yeszhanov A.B., Zhatkanbayeva Z.K., Chizhik S.A., Zdorovets M.V., Güven O., Korolkov I.V. (2024). Preparation and Application of Stimuli-Responsive PET TeMs: RAFT Graft Block Copolymerisation of Styrene and Acrylic Acid for the Separation of Water-Oil Emulsions. RSC Adv..

[32] Zhou Y.N., Li J.J., Luo Z.H. (2016). Toward Efficient Water/Oil Separation Material: Effect of Copolymer Composition on PH-Responsive Wettability and Separation Performance. AIChE J..

[33] Yang J., Loh X.J., Tan B.H., Li Z. (2019). PH-Responsive Poly(Dimethylsiloxane) Copolymer Decorated Magnetic Nanoparticles for Remotely Controlled Oil-in-Water Nanoemulsion Separation. Macromol. Rapid Commun..

[34] Muslimova I.B., Zhatkanbayeva Z.K., Omertasov D.D., Melnikova G.B., Yeszhanov A.B., Güven O., Chizhik S.A., Zdorovets M.V., Korolkov I.V. (2023). Stimuli-Responsive Track-Etched Membranes for Separation of Water-Oil Emulsions. Membranes.

[35] Ma H., Cameron A. (2023). Dual-Responsive Polymers Synthesized via RAFT Polymerization for Controlled Demulsification and Desorption. J. Polym. Res..

[36] Chen J., Yu Q., Wang M., Liu D., Dong L., Cui Z., He B., Li J., Yan F. (2024). Superhydrophilic/Underwater Superoleophobic PVDF Ultrafiltration Membrane with PH-Responsive Self-Cleaning Performance for Efficient Oil-Water Separation. Sep. Purif. Technol..

[37] Chen Q., Deng X., An Z. (2014). PH-Induced Inversion of Water-in-Oil Emulsions to Oil-in-Water High Internal Phase Emulsions (HIPEs) Using Core Cross-Linked Star (CCS) Polymer as Interfacial Stabilizer. Macromol. Rapid Commun..

[38] Yeszhanov A.B., Korolkov I.V., Dosmagambetova S.S., Zdorovets M.V., Güven O. (2021). Recent Progress in the Membrane Distillation and Impact of Track-Etched Membranes. Polymers.

[39] Korolkov I.V., Narmukhamedova A.R., Melnikova G.B., Muslimova I.B., Yeszhanov A.B., Zhatkanbayeva Z.K., Chizhik S.A., Zdorovets M.V. (2021). Preparation of Hydrophobic PET Track-Etched Membranes for Separation of Oil–Water Emulsion. Membranes.

[40] Guerre M., Wahidur Rahaman S.M., Améduri B., Poli R., Ladmiral V. (2016). RAFT Synthesis of Well-Defined PVDF-b-PVAc Block Copolymers. Polym. Chem..

[41] Keddie D.J. (2014). A Guide to the Synthesis of Block Copolymers Using Reversible-Addition Fragmentation Chain Transfer (RAFT) Polymerization. Chem. Soc. Rev..

[42] Monteiro M.J. (2005). Modeling the Molecular Weight Distribution of Block Copolymer Formation in a Reversible Addition-Fragmentation Chain Transfer Mediated Living Radical Polymerization. J. Polym. Sci. A Polym. Chem..

[43] Shu H., Wang C., Yang L., Sun D., Song C., Zhang X., Chen D., Ma Y., Yang W. (2024). Preparation of Multifunctional PET Membrane and Its Application in High-Efficiency Filtration and Separation in Complex Environment. J. Hazard. Mater..

[44] Shi Q., Su Y., Ning X., Chen W., Peng J., Jiang Z. (2010). Graft Polymerization of Methacrylic Acid onto Polyethersulfone for Potential PH-Responsive Membrane Materials. J. Memb. Sci..

[45] Romero-Fierro D.A., Camacho-Cruz L.A., Bustamante-Torres M.R., Hidalgo-Bonilla S.P., Bucio E. (2022). Modification of Cotton Gauzes with Poly(Acrylic Acid) and Poly(Methacrylic Acid) Using Gamma Radiation for Drug Loading Studies. Radiat. Phys. Chem..

[46] Henkel R.C. (2014). Der Einfluss Der UV-Initiierten RAFT-Polymerisation Auf Die Strukturen Und Eigenschaften von Polymernetzwerken. https://ediss.uni-goettingen.de/handle/11858/00-1735-0000-0023-98E2-6.

[47] Han S., Wu J., Zhang Y., Lai J., Chen Y., Zhang L., Tan J. (2021). Utilization of Poor RAFT Control in Heterogeneous RAFT Polymerization. Macromolecules.

[48] Hu Y., Hou Q., Liu H., Ye X. (2024). One-Pot, Surfactant-Free Synthesis of Poly(Styrene-N,N′-Methylenebis(2-Propenamide)-Acrylic Acid) and Poly(Styrene-N,N′-Methylenebis(2-Propenamide)-Methacrylic Acid) Microspheres for Adsorptive Removal of Heavy Metal Ions. Colloids Surf. A Physicochem. Eng. Asp..

[49] Korolkov I.V., Yeszhanov A.B., Zdorovets M.V., Gorin Y.G., Güven O., Dosmagambetova S.S., Khlebnikov N.A., Serkov K.V., Krasnopyorova M.V., Milts O.S. (2019). Modification of PET Ion Track Membranes for Membrane Distillation of Low-Level Liquid Radioactive Wastes and Salt Solutions. Sep. Purif. Technol..

[50] Safarpour M., Hosseinpour S., Haddad Irani-nezhad M., Orooji Y., Khataee A. (2022). Fabrication of Ti_2_SnC-MAX Phase Blended PES Membranes with Improved Hydrophilicity and Antifouling Properties for Oil/Water Separation. Molecules.

[51] Feng L., Gao Y., Yin W., Gao B., Yue Q. (2024). Multi-Functional Membrane with Double-Barrier and Self-Cleaning Ability for Emulsion Separation: Fouling Model and Long-Term Operation. J. Memb. Sci..

[52] Liu Y., Wang Y., Zhang T.C., Ouyang L., Yuan S. (2024). Switchable Superlyophobic PAN@Co-MOF Membrane for on-Demand Emulsion Separation and Efficient Soluble Dye Degradation. Sep. Purif. Technol..

[53] Li D., Lin J., An Z., Li Y., Zhu X., Yang J., Wang Q., Zhao J., Zhao Y., Chen L. (2020). Enhancing Hydrophilicity and Comprehensive Antifouling Properties of Microfiltration Membrane by Novel Hyperbranched Poly(N-Acryoyl Morpholine) Coating for Oil-in-Water Emulsion Separation. React. Funct. Polym..

