# Supplementary material for: Preparation of pH-Responsive PET TeMs by Controlled Graft Block Copolymerisation of Styrene and Methacrylic Acid for the Separation of Water–Oil Emulsions
Source: Polymers (Basel). 2025 Aug 14;17(16):2221. doi: 10.3390/polym17162221 (PMC12389503; doi:10.3390/polym17162221)
Supplement: Supplementary file 1 [file polymers-17-02221-s001.zip › polymers-3740950-supplementary.pdf]

Supplementary Materials

Preparation of pH-Responsive PET TeMs by Controlled Graft Block Copolymerisation of Styrene and Methacrylic Acid for the Separation of Water-Oil Emulsions

Indira B. Muslimova [1,2,*], Dias D. Omertassov [1,2], Nurdaulet Zhumanazar [1], Nazerke Assan [2], Zhanna K. Zhatkanbayeva [2] and Ilya V. Korolkov [1,2,*]

[1] The Institute of Nuclear Physics, Ibragimov Str. 1, Almaty 050032, Kazakhstan; dias2101@mail.ru (D.D.O.); nurdauletzhumanazar@gmail.com (N.Z.);
[2] L.N. Gumilyov Eurasian National University, Satpaev Str., 2, Astana 010000, Kazakhstan; nazerke.asanova65@mail.ru (N.A.); zhatkanbayeva_zhk@enu.kz (Z.K.Z.)
* Correspondence: i.muslimova@inp.kz (I.B.M.); i.korolkov@inp.kz (I.V.K.)

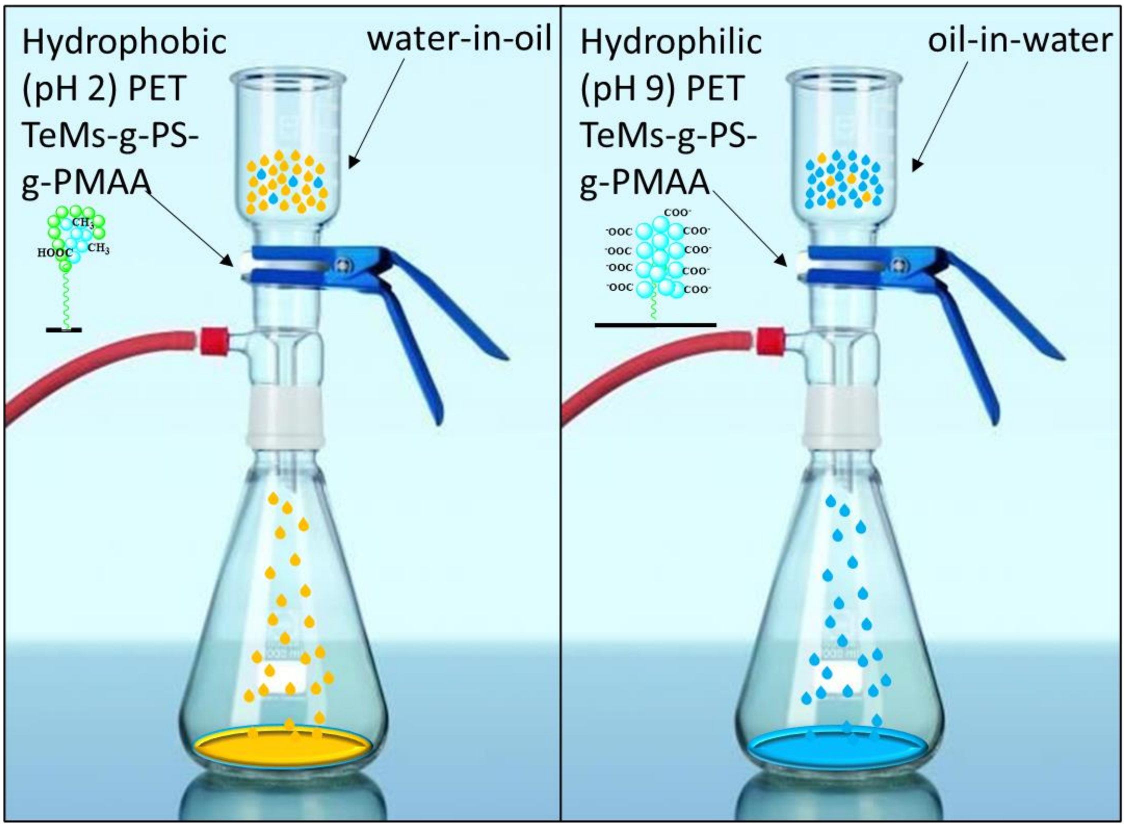

Figure S1. Dead-end vacuum filtration system used for membrane testing

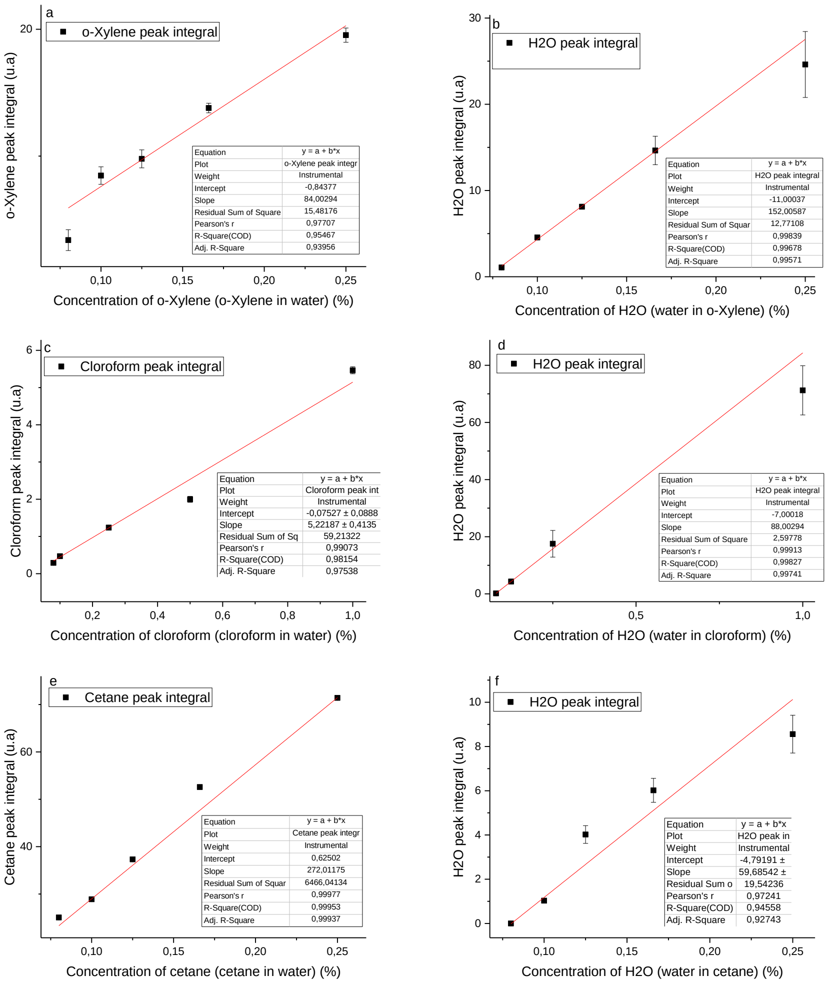

Figure S2. ^{1}H NMR calibration curves for determining dispersed phase concentrations (Cp) in permeate, used for calculating membrane rejection degree (R) in emulsion separation: o-xylene in water (a), water-in-o-xylene (b), cloroform-in-water (c), water-in-chloroform (d), cetane-in-water (e) and water-in-cetane (f)

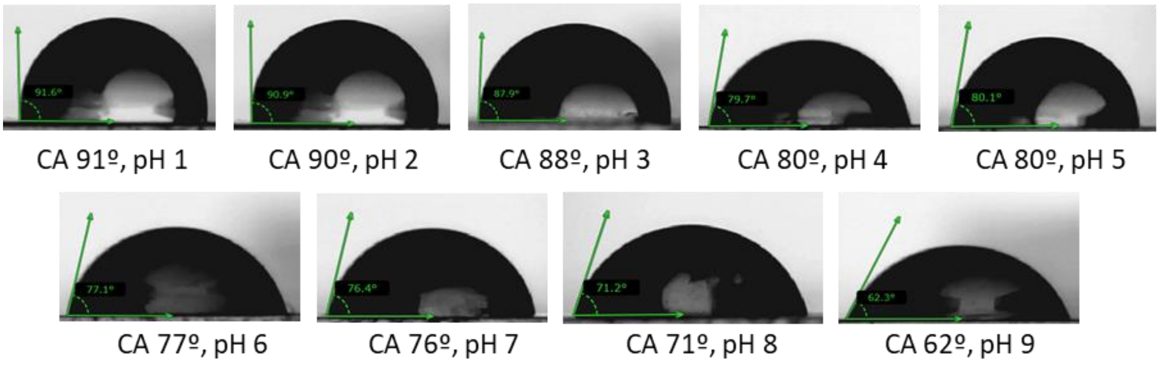

Figure S3. CA of PET TeMs-g-PS-g-PMAA (DG_{PMAA} 1.1±0.06%) as a function of pH (1–9), used to select optimal pH values for emulsion separation experiments

Tabel S1. Stability of the emulsions before separation

Emulsion	Time after emulsion formation, min	Avarage droplet size (according to DLS), um
	5	0.4
Chloroform-in-water	15	5.3
	40	5.6
	5	0.6
o-Xylene-in-water	15	4.4
	40	4.5
	5	2.1
Cetane-in-water	15	4.2
	40	4.5
	5	0.4
Water-in-chloroform	15	0.7
	40	0.8
	5	0.2
Water-in-*o*-xylene	15	1.3
	40	1.7
	5	0.4
Water-in-cetane	15	6.3
	40	8.6

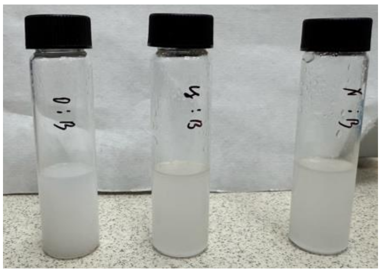

1 min

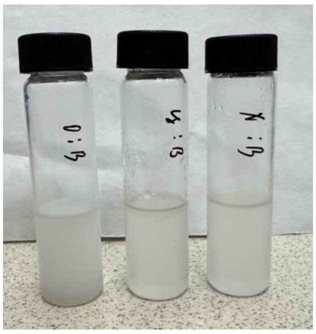

5 min

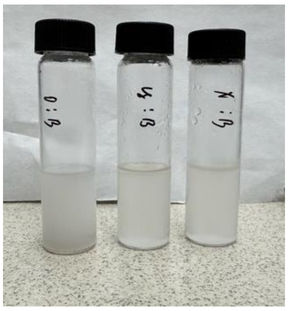

15 min

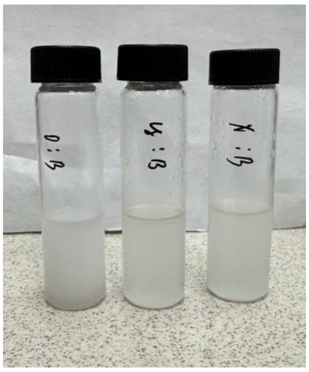

30 min

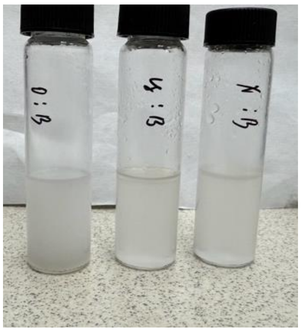

60 min

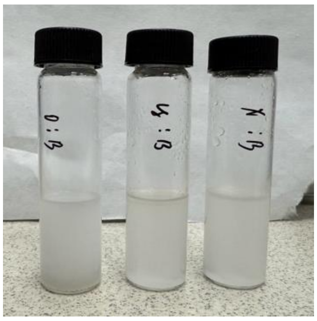

120 min

Figure S4. Pictures of emulsions during time (before filtrations) as example for *o*-Xylene-in-water, Cetane-in-water and Chloroform-in-water (prepared by Ultra-Turrax IKA disperser)

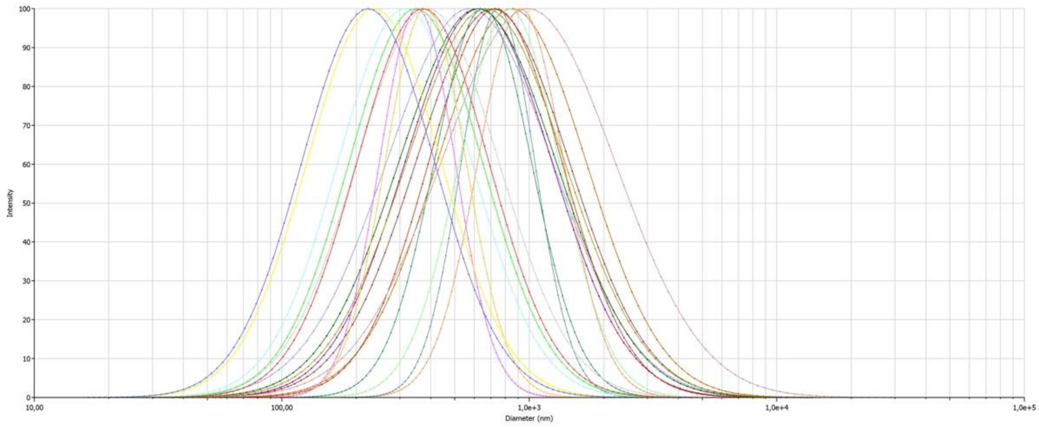

Water-in-chloroform

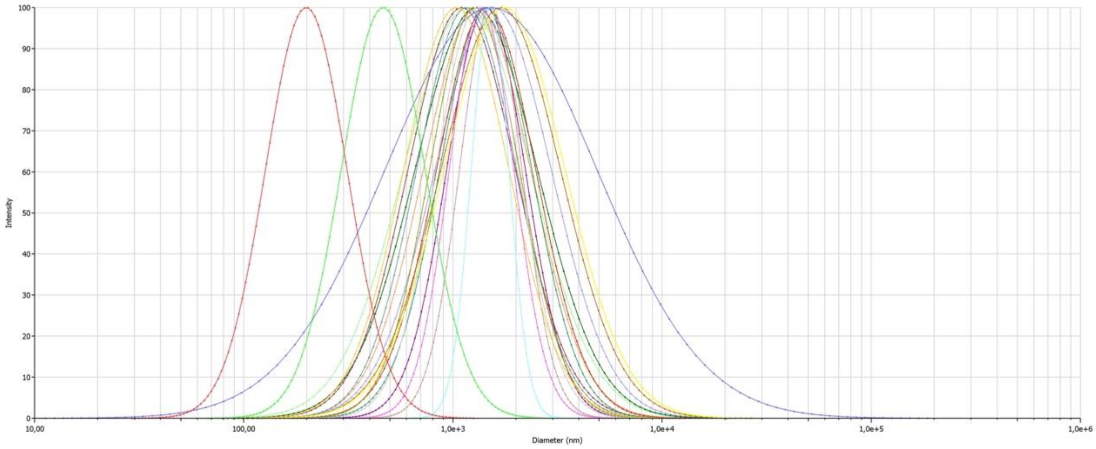

Water-in-*o*-xylene

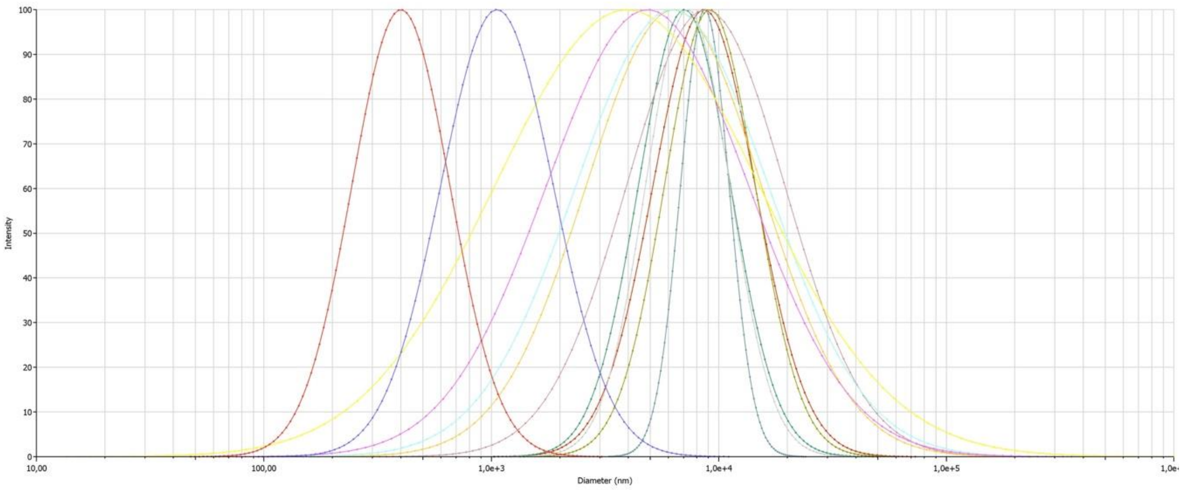

Water-in-cetane

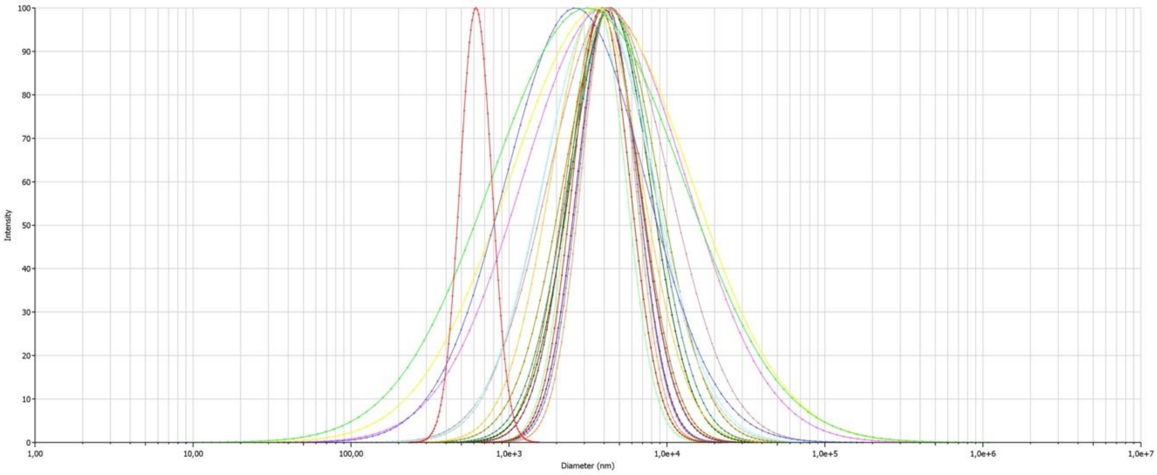

o-Xylene-in-water

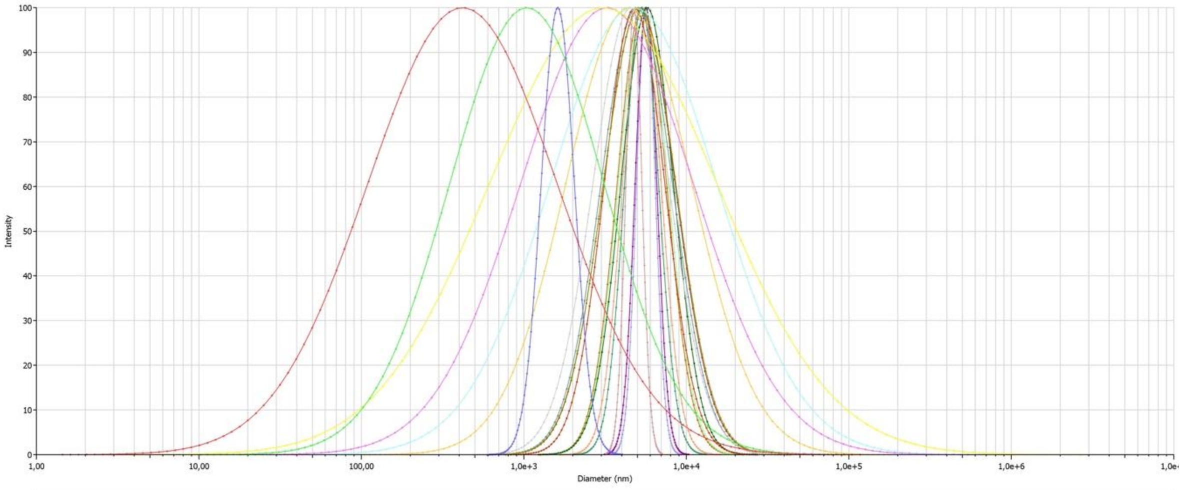

Chloroform-in-water

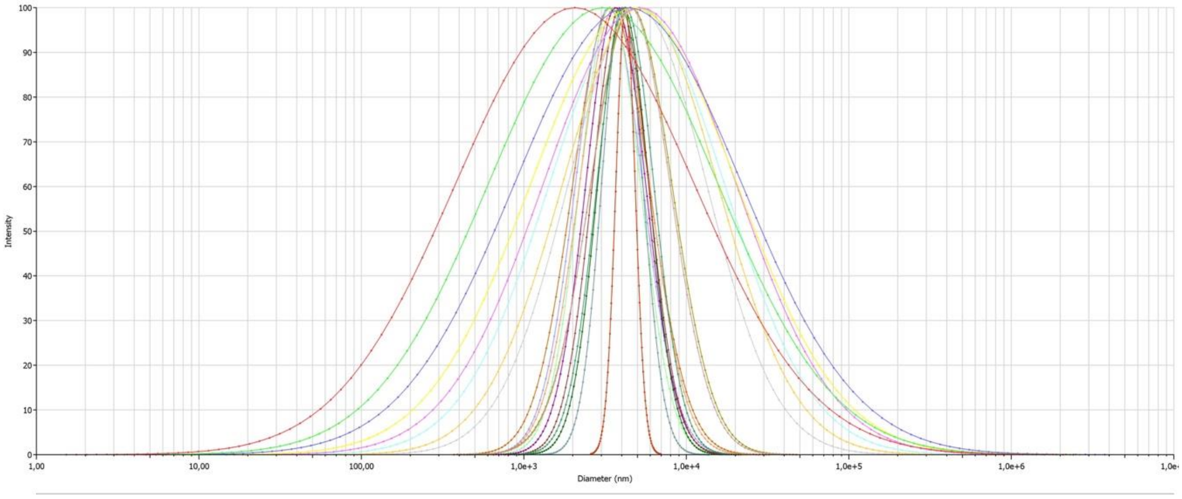

Cetane-in-water

Figure S5. DLS results for each emulsion (stability before separation during 5 min to 40 min with the step of 105 sec)

(a)

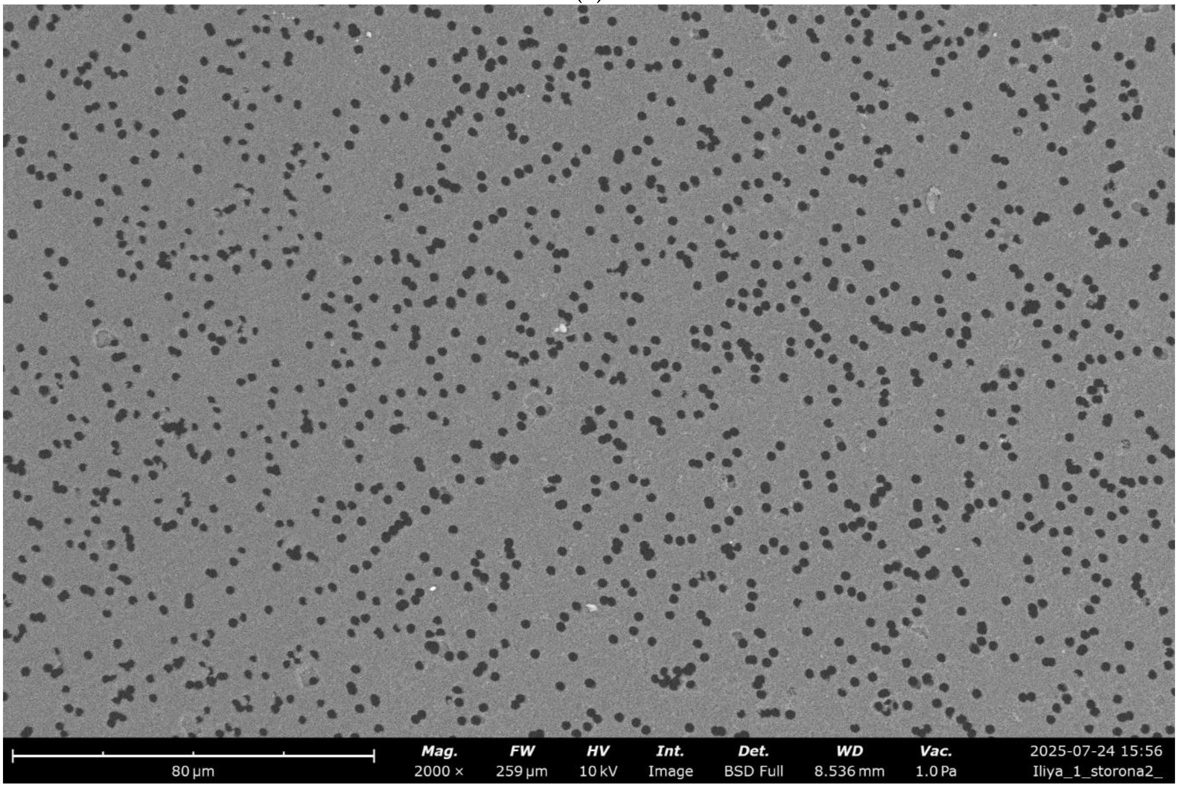

(b)

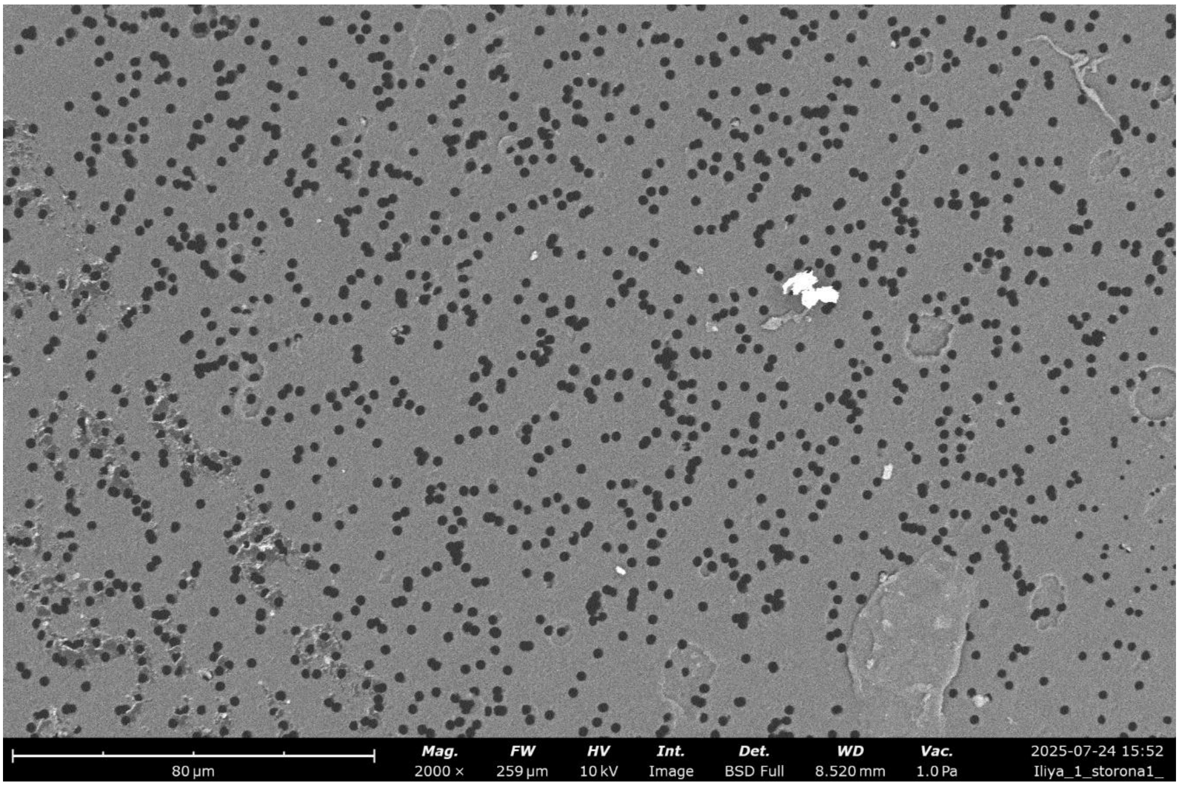

Figure S6. SEM images of PET TeMs-g-PS-g-PMAA membranes before (a) and after (b) five separation cycles showing the development of surface fouling
